# Microbicidal actives with virucidal efficacy against SARS-CoV-2 and other beta- and alpha-coronaviruses and implications for future emerging coronaviruses and other enveloped viruses

**DOI:** 10.1038/s41598-021-84842-1

**Published:** 2021-03-11

**Authors:** M. Khalid Ijaz, Raymond W. Nims, Sifang Steve Zhou, Kelly Whitehead, Vanita Srinivasan, Tanya Kapes, Semhar Fanuel, Jonathan H. Epstein, Peter Daszak, Joseph R. Rubino, Julie McKinney

**Affiliations:** 1 Reckitt Benckiser LLC, Global Research and Development for Lysol and Dettol, One Philips Parkway, Montvale, NJ 07645 USA; 2 Medgar Evers College of the City University of New York (CUNY), 1650 Bedford Ave, Brooklyn, NY 11225 USA; 3 RMC Pharmaceutical Solutions, Inc, 1851 Lefthand Circle, Suite A, Longmont, CO 80501 USA; 4 Microbac Laboratories, Inc, 105 Carpenter Drive, Sterling, VA 20164 USA; 5 EcoHealth Alliance, 520 Eighth Avenue, Suite 1200, New York, NY 10018-6507 USA

**Keywords:** Microbiology, Diseases

## Abstract

Mitigating the risk of acquiring coronaviruses including SARS-CoV-2 requires awareness of the survival of virus on high-touch environmental surfaces (HITES) and skin, and frequent use of targeted microbicides with demonstrated efficacy. The data on stability of infectious SARS-CoV-2 on surfaces and in suspension have been put into perspective, as these inform the need for hygiene. We evaluated the efficacies of formulated microbicidal actives against alpha- and beta-coronaviruses, including SARS-CoV-2. The coronaviruses SARS-CoV, SARS-CoV-2, human coronavirus 229E, murine hepatitis virus-1, or MERS-CoV were deposited on prototypic HITES or spiked into liquid matrices along with organic soil loads. Alcohol-, quaternary ammonium compound-, hydrochloric acid-, organic acid-, *p*-chloro-*m*-xylenol-, and sodium hypochlorite-based microbicidal formulations were evaluated per ASTM International and EN standard methodologies. All evaluated formulated microbicides inactivated SARS-CoV-2 and other coronaviruses in suspension or on prototypic HITES. Virucidal efficacies (≥ 3 to ≥ 6 log_10_ reduction) were displayed within 30 s to 5 min. The virucidal efficacy of a variety of commercially available formulated microbicides against SARS-CoV-2 and other coronaviruses was confirmed. These microbicides should be useful for targeted surface and hand hygiene and disinfection of liquids, as part of infection prevention and control for SARS-CoV-2 and emerging mutational variants, and other emerging enveloped viruses.

## Introduction

The guidance provided by the U.S. Centers for Disease Control and Prevention (CDC)^1^, the World Health Organization (WHO)^2,3^, and other regional centers for disease prevention and control discuss the infection prevention and control (IPAC) strategies of most utility in dealing with the COVID-19 pandemic. These would appear to be social distancing, the wearing of face masks, and the use of microbicides for hand hygiene and for sanitization of high-touch environmental surfaces (HITES)^4^. The latter include, but are not limited to, the toilet, bathroom and kitchen sinks, food preparation surfaces, door knobs, toys, desk tops, coins and paper currency, cell phones and other small electronic devices, automatic teller machines, and shopping carts, etc.)^5^. The use of surface- and hand-hygiene agents should be informed by knowledge of the likelihood of severe acute respiratory syndrome coronavirus-2 (SARS-CoV-2) contamination of such HITES. Other important factors include the persistence (survival or stability) of infectious virus released within bodily secretions/excretions from infected individuals on HITES, the likelihood of transfer of virus from HITES to hands, the persistence (survival) of virus on the skin once transferred, and the hierarchy of susceptibility of virus to microbicides. The hygiene agents should be targeted to HITES and to skin, and should be applied with appropriate frequency. The microbicides should be used as instructed, and applied using contact times that have been demonstrated empirically to have adequate virucidal efficacy. Reports of improper use of cleaning agents have surfaced^6^. As a result, scientists at the U.S. CDC have stressed that “Public messaging should continue to emphasize evidence-based, safe cleaning and disinfection practices to prevent SARS-CoV-2 transmission in households, including hand hygiene and cleaning and disinfection of high-touch surfaces”^6^.

On the basis of the known susceptibility of lipid-enveloped viruses, such as the coronaviruses, to microbicides^7–9^, reduction of the burden of infectious SARS-CoV-2 and other emerging coronaviruses remaining on HITES should readily be achieved through use of a variety of commonly-employed formulated microbicides. This paper is intended to complement and expand on a previous report on the virucidal efficacy of a number of commercially available formulated microbicides^10^. We have now included antiseptic liquids, disinfectant wipes, disinfectant liquids, disinfectant sprays, and sodium hypochlorite against SARS-CoV-2 and other coronaviruses tested on inanimate non-porous surfaces per ASTM E1053-20^11^. In addition, we have also tested a bar soap, an antiseptic liquid, a surface cleanser, two hand sanitizing gels, a liquid handwash, two foaming handwashes, and a toilet bowl cleanser for virucidal efficacy against SARS-CoV-2 and human coronavirus 229E in suspension studies conducted per ASTM E1052-20^12^ or EN 14,476:2013 + A2:2019^13^. We have expanded the evaluation to include additional beta-coronaviruses, including murine hepatitis virus-1 (MHV-1), severe acute respiratory syndrome coronavirus (SARS-CoV), and Middle East respiratory syndrome coronavirus (MERS-CoV), and the alpha-coronavirus human coronavirus 229E (HCoV-229E). In addition, we have developed the theme of persistence (survival) of infectious SARS-CoV-2, once deposited or spilled and then dried on HITES or on skin. This persistence information informs the need for targeted surface- and hand-hygiene applied at an appropriate frequency. In addition, we discuss the risk associated with incomplete inactivation of coronaviruses that subsequently might be released to the environment. This information informs the need for properly formulated microbicidal actives that may be used to decontaminate SARS-CoV-2 and other coronaviruses suspended in liquid matrices, such as pathophysiological secretions/excretions, residual virus in pre-soaked wipes following use for sanitizing surfaces, and waste handwash rinse water.

## Results

### Survival of SARS-CoV-2 on inanimate surfaces (prototypic HITES) and animate surfaces (swine skin)

Several studies of the survival (persistence of infectivity) of SARS-CoV-2 experimentally dried on prototypic HITES, or added to human secretions/excretions or skin have been reported in the recent literature^14–22^. These studies have evaluated the recovery of infectious SARS-CoV-2 from hard non-porous surfaces (such as steel and glass), from relatively porous surfaces (such as wood and cardboard), or from skin or within bodily secretions/excretions. The data sets have included the determined infectious SARS-CoV-2 titer at various times following deposition and drying on the prototypic HITES or after being added to skin or bodily secretions. The survival half-life values (t½, time required to reduce the SARS-CoV-2 titer by one-half) have been provided in the cited literature or were, in some cases^16,18^, calculated here from reported raw data to reflect biphasic or monophasic decay values, as appropriate to the reported data sets.

These viral persistence data (Table 1) indicate that, once deposited on prototypic HITES or swine skin, or when added to human secretions/excretions, infectious SARS-CoV-2 is recoverable from the surfaces/suspensions for hours to weeks. Survival half-life on surfaces was found to depend a number of factors. These include: (1) the type and porosity of the surface (including skin), (2) the presence and type of organic matrix in which the virus is suspended at the time of deposition onto the surface, (3) time, and (4) environmental factors such as temperature and relative humidity (RH). In suspension inactivation studies, relatively short half-lives (1.9 to 3.7 h) were observed in human sputum, mucus, or fecal suspensions^17,20^. A longer half-life (16 h) was determined in human urine^20^ for SARS-CoV-2. While some of the studies^14,17,19,21^ examined the impact of temperature or RH on viral persistence, for the most part, the studies evaluated virus survival at ambient temperature and RH, and we have reported only these data in Table 1.

**Table 1:** Literature values for terminal survival half-life (t½) of SARS-CoV-2 on prototypic HITES, on skin, or in suspension.

Prototypic fomite/suspension	Organic load	Temperature (RH)	Survival t½ (h)	Time needed for 1 log_10_ reduction in titer (h)	Time needed to decrease viral burden below MID (h)^a^	References
Plastic	None	21–23 °C (40%)	6.8	23	91	^15^
	None	22 °C (65%)	11	37	147	^14^
	None	25–27 °C (35%)	16	53	213	^20^
	None	19–21 °C (45–55%)	35	115	460	^16^
	10 mg/mL BSA	19–21 °C (45–55%)	24	79	316	^16^
	Human sputum	21 °C (40%)	3.1	10	40	^17^
	Human mucus	21 °C (40%)	3.1	10	40	^17^
	Tripartite soil	20 °C (35–40%)	38	130	520	^18^
Stainless steel	None	21–23 °C (40%)	5.6	19	75	^15^
	None	22 °C (65%)	15	50	200	^14^
	None	25–27 °C (35%)	23	77	306	^20^
	Tripartite soil	20 °C (35–40%)	29	95	380	^18^
	Tripartite soil	20 °C (50%)	43	143	573	^21^
Aluminum	None	19–21 °C (45–55%)	0.33	1.1	4.4	^16^
	10 mg/mL BSA	19–21 °C (45–55%)	15	51	204	^16^
Glass	None	22 °C (65%)	4.8	16	64	^14^
	None	25–27 °C (35%)	22	73	293	^20^
	None	19–21 °C (45–55%)	7.0	23	93	^16^
	10 mg/mL BSA	19–21 °C (45–55%)	25	83	333	^16^
	Tripartite soil	20 °C (50%)	46	153	613	^21^
Wood	None	22 °C (65%)	0.71	2.4	9.5	^14^
	None	25–27 °C (35%)	21	70	280	^20^
Vinyl	Tripartite soil	20 °C (50%)	46	153	613	^21^
Copper	None	21–23 °C (40%)	0.77	2.6	10	^15^
Cardboard	None	21–23 °C (40%)	3.5	12	47	^15^
Liquid sputum	N/A	21 °C	1.9	6.3	25	^17^
Liquid mucus	N/A	21 °C	3.7	12	47	^17^
Swine skin	None	20–24 °C (40–50%)	3.5	12	47	^19^
Human feces (10% suspension)	None	25–27 °C (35%)	2.6	8.7	35	^20^
Human urine	None	25–27 °C (35%)	16	53	212	^20^

Abbreviations used: BSA, bovine serum albumin; MID, human minimal infectious dose; RH, relative humidity; t½, half-life.

^a^Calculated assuming an initial deposited virus burden of 1.0 × 10^6^ plaque-forming units (PFU) and an estimated human MID of 250 PFU (see “Methods” section).

Viral stability data, on their own, do not greatly inform the implications for virus transmission. In order to put these SARS-CoV-2 survival data into perspective, we have calculated and displayed in Table 1 the durations of time required to reduce an initial viral burden of 1.0 × 10^6^ plaque-forming units (PFU) to a level beneath an estimate of the minimal human infectious dose of 250 PFU. These calculations were performed as described in Table 1 on the basis of the stability values reported in the literature. The SARS-CoV-2 may remain infectious on different types of fomites, or in suspensions of various types of body discharges, for hours to days.

### Virucidal efficacy of an antiseptic liquid formulation for SARS-CoV-2 and other coronaviruses

We evaluated the virucidal efficacy of an antiseptic liquid, with *p*-chloro-*m*-xylenol (PCMX) as active ingredient tested at a final active concentration of ~ 0.12%, against various alpha- and beta-coronaviruses (MHV-1^23^, HCoV-229E, SARS-CoV, MERS-CoV, and SARS-CoV-2). The results of virucidal efficacy of PCMX for inactivating viruses dried on glass (Table 2) demonstrate complete inactivation of each tested coronavirus within 0.5 to 10 min contact time at ambient temperature. Complete inactivation of the infectious virus within the limits of detection of the assays used was observed in the case of each virus. The difference in log_10_ reduction noted relate more to limit of virus-detection of the assay than to differences in virucidal-efficacy. The suspension inactivation testing against SARS-CoV-2 also demonstrate complete inactivation within 1 min.

**Table 2:** Virucidal efficacy of an antiseptic liquid contining ~ 0.12% *p*-chloro-*m*-xylenol (PCMX) for inactivating a variety of coronaviruses in suspension or on a hard surface (glass).

	Coronavirus	Contact time	Temperature	Organic load	Log_10_ reduction
*Hard surface testing (glass)*					
MHV-1^23^	Beta-coronavirus	0.5 min	Ambient	None	≥ 4.2, ≥ 4.5, ≥ 4.5, ≥ 4.5, ≥ 4.5^a^
HCoV-229E	Alpha-coronavirus	10 min	20 ± 2 °C	10 FBS	≥ 4.0
SARS-CoV	Beta-coronavirus	5 min	20 ± 1 °C	5% FBS	≥ 6.0, ≥ 6.0^b^
MERS-CoV	Beta-coronavirus	5 min	Ambient	5% BSA	≥ 5.0, ≥ 5.2^a^
*Suspension testing*					
SARS-CoV-2	Beta-coronavirus	1 min	20 ± 1 °C	5% FBS	≥ 5.0

Abbreviations used: BSA, bovine serum albumin; FBS, fetal bovine serum; HCoV-229E, human coronavirus strain 229E; MERS-CoV, Middle Eastern respiratory syndrome virus; MHV-1, murine hepatitis virus 1; PCMX, *p*-chloro-*m*-xylenol; SARS-CoV, severe acute respiratory syndrome coronavirus; SARS-CoV-2, severe acute respiratory syndrome coronavirus 2.

^a^The values are for technical replicates.

^b^The values are for independent lots.

### Virucidal efficacy of formulated microbicidal actives for SARS-CoV-2 and other coronaviruses experimentally deposited on glass

The virucidal efficacy of a variety of formulated microbicidal actives was tested per ASTM E1053-20 Standard^11^ using infectious SARS-CoV-2 and other beta- and alpha-coronaviruses dried on a glass surface in the presence of an organic load at ambient temperature (20 ± 1 °C). The results are displayed in Table 3. Virucidal efficacy displayed by the microbicides against the two beta-coronaviruses and the alpha-coronavirus were similar, as expected on theoretical grounds^7–9,24^. Contact times of 0.5 to 10 min led to ≥ 3.0 to ≥ 6.0 log_10_ reduction in coronavirus titer in the case of each of the formulated microbicidal actives evaluated, including PCMX, QAC, organic acids, ethanol/QAC, and sodium hypochlorite. Lot-to-lot variability of virucidal efficacy of the formulated microbicidal actives was minimal.

**Table 3:** Virucidal efficacy of formulated microbicides tested per ASTM E1053-20 Standard against HCoV-229E, SARS-CoV, or SARS-CoV-2 dried on a glass surface in the presence of an organic load.

Product type	Active ingredient concentration		Temperature (°C)	Contact time (min)	Organic load	Log_10_ reduction in infectious titer achieved^a^		
	In product	Tested				Alpha-coronavirus	Beta-coronavirus	
						HCoV-229E	SARS-CoV	SARS-CoV-2
Antiseptic liquid	PCMX (4.7% w/v)	0.125% w/v (tested at 1:40 of supplied)	20 ± 1	5, 10^e^	5, 10% FBS^e^	≥ 4.0	≥ 6.0, ≥ 6.0	NT
Disinfectant wipes	QAC^b^ (0.19% w/w)	0.19% w/w (tested as supplied)	20 ± 1	1.75	5% FBS	≥ 6.0	≥ 5.8	≥ 3.5, ≥ 3.5, ≥ 3.5
	Citric acid (2.4% w/w)	2.4% w/w (tested as supplied)	20 ± 1	0.5	5% FBS	≥ 4.3, ≥ 4.3	≥ 3.0, ≥ 3.0	≥ 3.0, ≥ 3.0, ≥ 3.0
Disinfectant spray	Ethanol (50% w/w)/ QAC^c^ (0.082% w/w)	50% w/w ethanol, 0.082% w/w QAC^c^ (tested as supplied)	20 ± 1	0.5, 1.75^f^	5% FBS	≥ 5.5, ≥ 5.5,	NT	≥ 4.6, ≥ 4.7, ≥ 4.5
Dilutable cleaner	QAC^b^ (2.9% w/w)	0.0916% (tested at 1:32 of supplied)	20 ± 1	5	5% FBS	≥ 3.5, ≥ 3.5	≥ 4.8, ≥ 4.8	NT
RTU cleaner	QAC^d^ (0.092% w/w)	0.092% (tested as supplied)	20 ± 1	2	5% FBS	≥ 3.3, ≥ 3.3	≥ 3.8, ≥ 3.8	≥ 4.0, ≥ 4.0, ≥ 4.0

In all cases, one technical replicate was performed per data point.

Abbreviations used: FBS, fetal bovine serum; HCoV-229E, human coronavirus strain 229E; NT, not tested; PCMX, *p*-chloro-*m*-xylenol; QAC, quaternary ammonium compound; RTU, ready to use; SARS-CoV, severe acute respiratory syndrome coronavirus; SARS-CoV-2, severe acute respiratory syndrome coronavirus 2; w/v, weight-to-volume; w/w, weight-to-weight.

^a^Where multiple values are displayed, this reflects the testing of multiple independent lots of the formulated microbicide.

^b^Alkyl (50% C14, 40% C12, 10% C16) dimethyl benzyl ammonium chloride.

^c^Alkyl (50% C14, 40% C12, 10% C16) dimethyl benzyl ammonium saccharinate.

^d^Alkyl (67% C12, 25% C14, 7% C16, 1% C8-C10-C18) dimethyl benzyl ammonium chloride; Alkyl (50% C14, 40% C12, 10% C16) dimethyl benzyl ammonium chloride.

^e^The 10-min contact time and 10% FBS load were used in the HCoV-229E study.

^f^The 0.5-min contact time was used for the HCoV-229E study and the 1.75-min contact time was used for the SARS-CoV-2 study.

### Virucidal efficacy of formulated microbicidal actives for SARS-CoV-2 and other coronaviruses evaluated in suspension

The virucidal efficacy of a variety of formulated microbicidal actives was tested per ASTM-E1052-20^12^ (handwash agents; Table 4) or EN 14,476:2013 + A2:2019^13^ (hand sanitizers, antiseptic liquids and sprays, surface cleaners, toilet cleaners, etc.; Table 5), using infectious SARS-CoV-2 and HCoV-229E in suspension studies. Contact times of 0.5 to 1 min at ~ 37 °C led to ≥ 3.0 to ≥ 3.6 log_10_ reduction in coronavirus titer in the case of the handwash agents containing actives such as PCMX, salicylic acid, or benzalkonium chloride (Table 4). Contact times of 0.5 to 5 min at ambient temperature led to ≥ 4.0 to ≥ 5.5 log_10_ reduction in coronavirus titer in the case of each of the actives-based formulations evaluated in the EN 14,476 studies, including PCMX, benzalkonium chloride, organic and inorganic acids, ethanol, and sodium hypochlorite (Table 5).

**Table 4:** Virucidal efficacy of formulated microbicidal actives tested per ASTM-E1052-20 Standard against HCoV-229E or SARS-CoV-2 in suspension inactivation studies.

Product type	Active ingredient concentration		Temperature (°C)	Contact time (min)	Organic load	Log_10_ reduction in infectious titer achieved	
	In product	Tested				Alpha-coronavirus	Beta-coronavirus
						HCoV-229E	SARS-CoV-2
Bar soap	PCMX (0.090% w/w)	0.014% w/w (tested at 1:6.25 of supplied)	37 ± 1	0.5, 1^a^	5% FBS	≥ 3.3	≥ 4.1
Liquid gel handwash	Salicylic acid (0.10% w/w)	0.025% w/w (tested at 1:4 of supplied)	37 ± 1	0.5, 1^a^	5% FBS	≥ 3.6, ≥ 3.6,	≥ 3.6
Foaming handwash	Benzalkonium chloride (0.10% w/w)	0.025% w/w (tested at 1:4 of supplied)	37 ± 1	1	5% FBS	≥ 3.3, ≥ 3.3	≥ 3.4
	Salicylic acid (0.09% w/w)	0.023% w/w (tested at 1:4 of supplied)	37 ± 1	0.5, 1^a^	5% FBS	≥ 3.5, ≥ 3.8	≥ 5.0

Where multiple cvalues are shown, these represent different technical replicates.

Abbreviations used: FBS, fetal bovine serum; HCoV-229E, human coronavirus strain 229E; PCMX, *p*-chloro-*m*-xylenol; SARS-CoV-2, severe acute respiratory syndrome coronavirus 2, w/w, weight-to-weight.

^a^A 1-min contact time was used for testing against HCoV-229E; an 0.5-min contact time was used for testing against SARS-CoV-2.

**Table 5:** Virucidal efficacy of formulated microbicidal actives tested per EN 14,476:2013 + A2:2019 Standard against HCoV-229E or SARS-CoV-2 in suspension inactivation studies.

Product type	Active ingredient concentration		Temperature (°C)	Contact time (min)	Organic load	Log_10_ reduction in infectious titer achieved	
	In product	Tested				Alpha-coronavirus	Beta-coronavirus
						HCoV-229E	SARS-CoV-2
*Hand hygiene agents*							
Antiseptic liquid	PCMX (4.7% w/v)	0.021% w/v (tested at 1:200 of supplied)	20 ± 1	5	Dirty^a^	≥ 5.2	≥ 4.7
Hand sanitizer gel	Ethanol (67% w/w)	53% w/w (tested at 1:1.25 of supplied)	20 ± 1	1	Dirty, clean^b^	≥ 5.4	≥ 4.2
	Citric acid (1.9% w/w), lactic acid (0.51% w/w)	1.5% w/w citric acid, 0.41% w/w lactic acid (tested at 1:1.25 of supplied)	20 ± 1	0.5, 1^c^	Clean^b^	≥ 5.2	≥ 4.7
*Surface hygiene agents*							
Surface cleaner	QAC^d^ (0.096% w/w)	0.077% w/w (tested at 1:1.25 of supplied)	20 ± 1	5	Dirty	NT	≥ 4.1
	Lactic acid (2.4% w/w)	1.9% (1:1.25 of supplied)	20 ± 1	5	Dirty	NT	≥ 5.5
Toilet bowl cleaner	Hydrochloric acid (6.9% w/w)	0.25% w/w (tested at 1:27 of supplied)	20 ± 1	0.5	Dirty	NT	≥ 4.1
Dilutable cleaner	Sodium hypochlorite (3.6% w/w)	0.14% w/w (tested at 1:26 of supplied)	20 ± 1	0.5	Clean^b^	NT	≥ 5.1
RTU cleaner	Benzalkonium chloride (0.56% w/w)	0.45% w/w (tested at 1:1.25 of supplied)	20 ± 1	5	Dirty	NT	≥ 4.5
Disinfectant spray	Ethanol (55% w/w)	Ethanol (44% w/w) used as supplied	20 ± 1	5	Dirty	≥ 4.0	≥ 4.1
Bathroom cleaner	Sodium hypochlorite (0.40% w/w)	0.32% w/w (tested at 1:1.25 of supplied)	20 ± 1	5	Dirty	NT	≥ 5.1

In all cases, one technical replicate was performed per data point.

Abbreviations used: BSA, bovine serum albumin; HCoV-229E, human coronavirus strain 229E; NT, not tested; PCMX, *p*-chloro-*m*-xylenol; RTU, ready to use; SARS-CoV-2, severe acute respiratory syndrome coronavirus 2, w/v, weight-to-volume, w/w, weight-to-weight.

^a^Dirty means 3 g/L BSA + 3 mg/L erythrocyte suspension.

^b^Clean means 0.3 g/L BSA, used for testing SARS-CoV-2.

^c^A 1-min contact time was used for testing against HCoV-229E; an 0.5-min contact time was used for testing against SARS-CoV-2.

^d^Alkyl dimethyl benzyl ammonium chloride (C12-16).

## Discussion

In response to the current outbreak of SARS-CoV-2 and the urgency around establishing evidence-based IPAC approaches, we and others have hypothesized that the virucidal efficacy of commonly used microbicides against this emerging coronavirus should be predictable on the basis of the known susceptibility of enveloped viruses in general to microbicides^7–9,24^. In this paper, we confirm the virucidal efficacy of a variety of formulated microbicidal actives against SARS-CoV-2 and a number of members of the *Coronaviridae* family (HCoV-229E, MERS-CoV, SARS-CoV, and MHV-1), indicating similar virucidal efficacy across members of the *Coronaviridae*. On the basis of these results, we predict that any potential future emerging coronaviruses or other emerging enveloped viruses also readily would be inactivated by these microbicides. The necessity for use of microbicides in IPAC for emerging viruses is informed by the routes of transmission of the viruses, the likelihood that they will be deposited on HITES, the expected duration of survival of the viruses on such HITES, and the frequency of recontamination of the HITES by infected persons.

The primary route of person-to-person transmission of SARS-CoV-2 is thought to involve respiratory droplets and aerosols, as reviewed in^25–27^, leading predominantly to a respiratory tract infection. Secondary (indirect) transmission of SARS-CoV-2 through contamination of HITES by droplets and respiratory aerosols or other patient secretions/excretions (bronchoalveolar fluid, sputum, mucus, blood, lacrimal fluid, semen, urine, and feces) also is thought to occur^25–28^. The indirect transmission pathway may be envisioned as a patient’s bodily fluids-HITES-hands-mucous membrane nexus. The relevance of this pathway is supported by experimental transmission studies in animal models^29^ and by the results of investigations of the contamination of HITES with SARS-CoV-2 RNA in healthcare settings^26,30–32^. The detection of infectious SARS-CoV-2 in patient feces^33,34^ and urine^35^, together with the data on survival of SARS-CoV-2 in fecal and urine suspensions^20,22^, suggest that a fecal/oral or fecal/respiratory route of transmission is possible. Zang et al.^36^, upon being unable to recover infectious SARS-CoV-2 from RNA-positive human fecal samples, have argued that the virus is rapidly inactivated by simulated human colonic fluid. This conclusion is not consistent, however, with the findings of Xiao et al*.*^33^ or Zhang et al*.*^34^, who were able to recover infectious SARS-CoV-2 from human feces, as reviewed in^37^, or with the reports of Liu et al.^20^ and Chan et al.^22^ that SARS-CoV-2 remains infectious for hours in human urine and fecal suspensions. The conclusion is also not consistent with results obtained for other coronaviruses, such as SARS-CoV and MERS-CoV^37^. These routes of transmission could involve direct transmission or indirect transmission involving the patient’s bodily fluids-HITES-hands-mucous membrane nexus mentioned above. The U.S. CDC has stated that “transmission of novel coronavirus to persons from surfaces contaminated with the virus has not been documented”, but nevertheless has provided guidance on surface disinfection^38^. The finding of SARS-CoV-2 RNA in untreated wastewater^39^ and sewage^40^, is suggestive of, but certainly not proof of, the possibility for survival of infectious virus within these human waste streams, as reviewed in^37,41,42^. Unfortunately, there are, to our knowledge, no data on the detection or persistence of infectious SARS-CoV-2 in wastewater, and this topic, therefore, remains a knowledge gap^37,41,42^. For the moment, on the basis of the reported survival of SARS-CoV-2 in human fecal suspensions and urine^20,22^, we assume the possibility of the contamination of wastewater streams with infectious SARS-CoV-2, and associated risk of virus dissemination through this route.

In order to inform the necessity of effective and frequent HITES decontamination during a virus pandemic, such as that being experienced currently with SARS-CoV-2, we have summarized and put into perspective the recent data on the survival of infectious SARS-CoV-2 on such surfaces under ambient conditions. Infectivity half-life values obtained from virus survival studies can be used to calculate the burden of infectious SARS-CoV-2 expected to remain on a surface after varying durations of time following initial virus deposit. This assumes, of course, that the initial virus load on the surface is known. There, unfortunately, is a paucity of empirical data on infectious SARS-CoV-2 burden (loads) on HITES in the literature thus far. The existing data consist primarily of measurements of nucleic acid burden on HITES. Findings from Matson et al.^17^ suggest that caution should be taken when making inferences regarding the possible presence of infectious virus on a surface based solely on RT-PCR detection of viral RNA. We very much share this concern.

The data on the survival of SARS-CoV-2 on surfaces^14–22,43^, like previous data obtained for other coronaviruses^43–51^, demonstrate that viral persistence (survival) on HITES is dependent upon: (1) the type of surface, (2) the presence and type of organic matrix in which the virus is suspended at the time of deposition and drying upon the surface, and (3) time. The survival data for SARS-CoV-2 dried on surfaces (Table 1) indicate that the virus remains infectious longer on hard non-porous surfaces, such as plastic and stainless steel, than on wood or cardboard. The presence of an organic load during drying of the virus typically results in increased half-life of SARS-CoV-2^16,18,21^. The result of Matson et al.^17^ that SARS-CoV-2 displayed a shorter half-life when dried on a surface in the presence of human sputum and mucus than when dried in a culture medium matrix was therefore unexpected, and requires confirmation. Temperature and relative humidity likely also play a role in the persistence of coronaviruses on HITES, although the data sets appearing in the literature specifically for SARS-CoV-2^14–22^ have primarily evaluated survival under ambient conditions. For survival dependence on temperature, see references^17,19,21,22,43^.

The viral persistence data indicate that infectious virus may remain on non-porous HITES for one or two weeks. The risk of acquiring a SARS-CoV-2 infection indirectly, through transfer of virus from a contaminated HITES to a susceptible mucous membrane through the intermediacy of the hands, therefore may remain for weeks after the initial surface contamination event. The requirement for frequent sanitization of HITES is driven by the possibility for recontamination of these environmental surfaces by infected persons^52^. Infectivity data addressing the frequency of recontamination of HITES with SARS-CoV-2 have not yet appeared in the literature, to our knowledge. This represents another knowledge gap. In the case of human coronavirus 229E, Bonny et al.^53^ demonstrated that infectious virus could be recovered from HITES (desktops and door knobs) in a university classroom that was cleaned daily with a commercial cleaning solution consisting of non-ionic and anionic surfactants. This result suggested the possibility of the frequent recontamination of the HITES, although the possible inadequacy of the daily cleaning regimen was not ruled out by the authors^53^.

The stability of SARS-CoV-2 in suspensions and on skin has also been investigated. The survival of the virus in human sputum and mucus is similar to that on porous surfaces (half-lives of 1.9 to 3.5 h, respectively)^17^. Survival on skin (3.5 h)^19^ is similar (Table 1). These half-life values indicate that the virus may remain infectious for days on HITES or skin following a contamination event, in the absence of hygiene interventions.

From the foregoing, it is apparent that there is a risk of indirect transmission of SARS-CoV-2 from contaminated HITES, via the intermediacy of hands. This risk may be mitigated through targeted hygiene interventions, including frequent surface hygiene as well as hand hygiene. The required frequency of surface hygiene interventions is dependent on the expected rate of recontamination of HITES by patients. This suggests that greater vigilance with respect to targeted hygiene practices is required in intensive care units and other contamination hot spots, as emphasized by Zhang^54^ and by the results of Wu et al.^55^.

The required efficacy of targeted hygiene agents (formulated microbicidal actives) for reducing the infectious titer of SARS-CoV-2 and other coronaviruses depends, in large part, on the burden of infectious virus on the surface or in the suspension being sanitized^56^ and the human minimal infectious dose (MID). Expected virucidal efficacy usually is expressed in terms of a minimal log_10_ reduction in viral titer to be achieved in standardized testing. For instance, the U.S. Environmental Protection Agency (EPA) specified in its 2012 disinfectant product guidance^57^ that “The product should demonstrate complete inactivation of the virus at all dilutions. If cytotoxicity is present, the virus control titer should be increased to demonstrate a ≥ 3 log_10_ reduction in viral titer beyond the cytotoxic level.” On the other hand, in the case of disinfectants that do not cause cytotoxicity in the cell-based infectivity assays used in virucidal efficacy testing, a 4-log_10_ reduction in viral titer is considered to demonstrate effectiveness. These EPA requirements were revised in the 2018 revision^58^. In the 2018 guidance, a valid test requires the following: (1) ≥ 4.8 log_10_ of infectivity per carrier be recovered from the dried virus control film; (2) ≥ 3 log_10_ reduction in titer is observed in the presence or absence of cytotoxicity; (3) if cytotoxicity is present, ≥ 3 log_10_ reduction in titer is observed beyond the cytotoxic level; and (4) cell controls (cells not spiked with virus) be negative for evidence of infectivity (i.e., viral cytopathic effect or plaques). The revised guidance therefore does not require that complete inactivation be observed at all dilutions for a product to be deemed effective.

**Figure 1:**
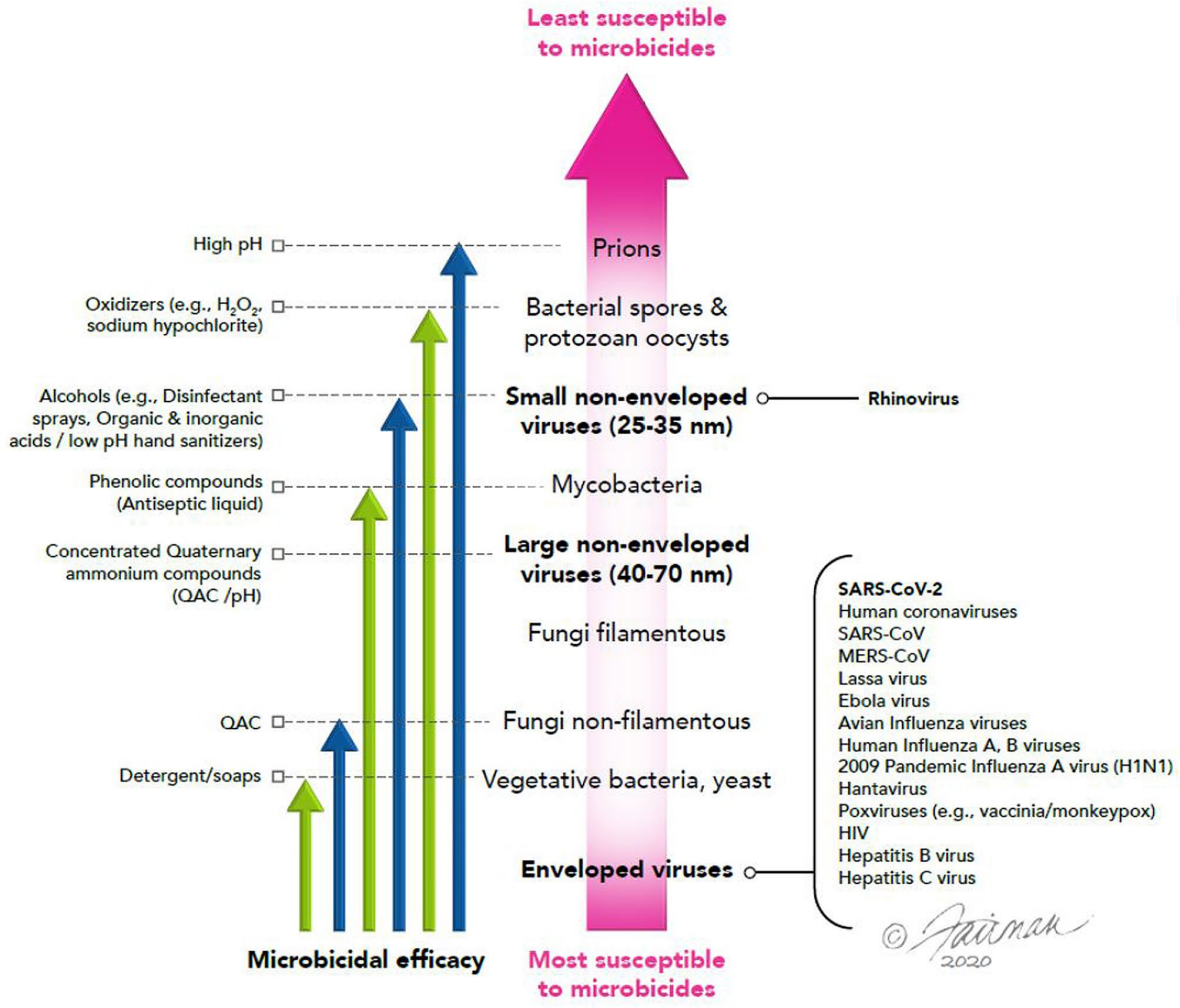
Heirarchy of susceptibility of pathogens to microbicidal active ingredients. Certain formulated microbicides may include combinations of active ingredients, resulting in synergistic virucidal efficacy greater than that displayed by the individual active ingredients (modified from Sattar^8^).

In our virucidal efficacy studies, a variety of formulated microbicidal actives displayed complete inactivation of the challenge coronaviruses (including SARS-CoV-2). The maximum log_10_ reduction values achieved depended on the limitations of the assays (namely, the maximum titer of virus applied to the test and the cytotoxicity associated with the formulated microbicidal active). In any event, log_10_ reduction values of ≥ 3 to ≥ 6 were obtained after relatively short contact times (i.e., 0.5 to 5 min.). These contact times are relevant for surface disinfection interventions and, notably, the contact times required for the hand hygiene agents evaluated (handwash agents and hand sanitizing gels) were ≤ 1 min. The active ingredients used in the formulated microbicidal agents evaluated in Tables 2, 3, 4, and 5 included agents with differing mechanisms of action^59^. These included lipid envelope-disrupting agents such as ethanol, QAC, detergents, and phenolics. Protein- and capsid-denaturing agents evaluated included ethanol, phenolics, sodium hypochlorite, inorganic and organic acids. The genome-degrading agents evaluated included ethanol, and sodium hypochlorite. Each of these types of microbicidal actives was expected, on the basis of the known susceptibility of pathogens to microbicides^7–9,24,59^ (Fig. 1), to display virucidal efficacy against lipid-enveloped viruses, including SARS-CoV-2 and other coronaviruses. This principle of the hierarchy of pathogen susceptibility has also been embraced by the U.S. EPA^60^. Our efficacy data presented herein confirm this, and indicate that the virucidal activities are approximately equivalent for a variety of alpha- and beta-coronaviruses. In addition, reviews and empirical reports of the efficacy of microbicides against SARS-CoV-2^10,22,48,49^ and other coronaviruses^23,44,46–49,61^ have confirmed the expected virucidal efficacy of a variety of microbicides against these viruses in surface disinfection studies. Efficacy of microbicides tested in suspension studies has been discussed in recent reviews and empirical reports of the efficacy of microbicides against SARS-CoV-2^10,14,22,49^ and other coronaviruses^44,46,48,49,61,62^. These also have confirmed the expected virucidal efficacy of a variety of microbicides against these viruses in suspension.

Taken together, these results imply that similar virucidal efficacies should be displayed by such microbicides against future emerging coronaviruses, including mutational variants (isolates) of SARS-CoV-2 such as the recently emerging and highly transmissible 20I/501Y.V1, VOC 202,012/01, or B.1.1.7 variant^63,64^. The virucidal efficacies would be expected^24^ to apply also to other emerging enveloped viruses, such as Ebola virus^65,66^, Lassa virus, Nipah virus, and influenza viruses such as the recently emerging G4 genotype H1N1 swine influenza virus^67^ and the variant influenza viruses (H1N1v, H3N2v, H1N2v) in humans^68^. The latter expectation is supported by our own unpublished data on influenza strains and by a recent literature review^61^. These are important conclusions, given that there is a likelihood of emergence of novel coronaviruses and other enveloped viruses in the future.

A large diversity of alpha- and beta-coronaviruses currently circulate in bat reservoirs^69^. These include the alpha-coronavirus, swine acute diarrhea syndrome coronavirus, which caused large-scale pig die-offs in southern China, and is able to infect human cells in the laboratory^70^. They also include a substantial diversity of SARS-related coronaviruses that include the progenitor lineages of SARS-CoV and SARS-CoV-2, primarily carried by horseshoe bats (*Rhinolophus* spp.)^71–76^. SARS-CoV emerged in 2002 within urban live animal markets in Guangdong, where a range of animal species being held there, as well as animal vendors themselves, were infected^77^. While the exact route of SARS-CoV-2 spillover from bats to humans is uncertain, evidence strongly implicates a similar live animal market as a site where infections were amplified, and where SARS-CoV-2 was identified on contaminated surfaces^71,78^. Subsequent clusters of COVID-19 have been reported in a large seafood market in Beijing, perhaps as a direct result of contamination of cold surfaces used to prepare food^79^. Thus, the role of food animals, food preparation, and contaminated surfaces in the spillover of these bat coronaviruses suggests a key role for disinfecting surfaces to mitigate spillover or early spread of novel bat coronaviruses.

There is also evidence that bat coronaviruses are transmitted regularly to people in southeast Asia, without involvement of wildlife consumption. First, diverse behaviors that bring people into contact with wildlife have been reported in South China^80,81^. Secondly, 2.79% of people sampled from communities living close to a bat cave in Yunnan, China, where SARS coronaviruses have been reported, were serologically positive for bat coronavirus immunoglobulin G (IgG)^82^. Extrapolating to rural communities across Southeast Asia where similar bats exist, and given that SARS-CoV IgG had a half-life of 2–3 years in SARS survivors, it is likely that hundreds of thousands of people are infected by novel bat coronaviruses each year. Surface disinfection and personal hygiene using agents that are effective at inactivating coronaviruses may, therefore, be critical to the control of the current SARS-CoV-2 pandemic, and in reducing the risk of future coronavirus spillover events.

Assuming, for the purpose of argument, that SARS-CoV-2 is transmitted from person-to-person in part through the patient’s bodily fluids-HITES-hands-mucous membrane nexus, what evidence do we have that implementing surface and hand hygiene interventions will mitigate risk of disseminating SARS-CoV-2? It is clear that face touching is a frequent human behavior^83^, suggesting that the indirect route of transmission occurring through the intermediacy of the hands is relevant, and highlighting the need for strict implementation of hand hygiene. This especially is the case when coming in contact with patients’ bodily fluids and when touching potentially contaminated HITES. Evidence has now been reported that disinfection can lead to reduction in dissemination of SARS-CoV-2 from infected persons to uninfected family members. For instance, Wang et al.^4^ reported that the daily use of chlorine- or ethanol-based disinfectants for household cleaning was 77% effective in reducing transmission of SARS-CoV-2 within the families investigated. Diarrhea as a symptom of the primary infected household member was also found to be a risk factor for transmission within families, informing the importance of sanitizing the toilets and the bathroom itself^4^.

The relatively high risk of the bathroom for deposition of SARS-CoV-2 from patients onto HITES was also highlighted in the study of Ding et al.^84^ In that study, frequency of sanitization of HITES was twice daily using a chlorine-releasing agent. Out of 107 surface samples and 46 air samples taken from a COVID-19 hospital ward, only eight were found to be positive for SARS-CoV-2 RNA. These included seven surface samples (two door handles, one toilet seat, one toilet seat cover, one bathroom washbasin tap handle, one bathroom ceiling exhaust louver, and one bathroom door handle) and one air sample (a corridor air sample)^84^.

Since it is not known yet whether infectious SARS-CoV-2 persists in wastewater streams^41,42^, we cannot address the question of whether hygiene interventions can reduce the infectious viral burden of such waste streams. This remains a significant knowledge gap that has yet to be closed^85^. There are data on the persistence of infectious virus in water for other coronaviruses, such as transmissible gastroenteritis virus, mouse hepatitis virus-1, and SARS-CoV, as reviewed in^42,43^. For the moment, the use of wastewater/sewage SARS-CoV-2 RNA data is limited to a biomarker for monitoring of ongoing COVID-19 outbreak intensity^39,40^. It is evident from the foregoing discussion, however, that targeted surface/hand hygiene, appropriately practiced under healthcare, community and home settings, can help to ensure that infectious SARS-CoV-2 is not released into the environment via wastewater streams.

## Conclusions

Indirect person-to-person transmission of SARS-CoV-2 from contaminated HITES through the intermediacy of the hand (i.e. through the patient’s bodily fluids-HITES-hands-mucous membrane nexus), is a relevant mechanism for dissemination of SARS-CoV-2 and the associated disease (COVID-19). Here, we have expanded on a previous report on the virucidal efficacy of a number of commercially available formulated microbicidal actives^10^ to now include antiseptic liquids, disinfectant wipes, disinfectant liquids, disinfectant sprays, and sodium hypochlorite for virucidal efficacy against SARS-CoV-2 and other coronaviruses on inanimate surfaces (prototypic HITES). In addition, we have also tested bar soap, antiseptic liquid, surface cleanser, hand sanitizing gels, liquid handwash, foaming handwash, and toilet bowl cleanser for efficacy against SARS-CoV-2 and human coronavirus 229E in suspensions intended to model animate surfaces/solutions (skin and bodily fluids). Each of these formulated microbicidal actives resulted in complete inactivation (≥ 3 to ≥ 6 log_10_ reduction in infectious titer within the limits of virus detection) of the challenge coronaviruses, including SARS-CoV-2. These surface- and personal-care hygiene agents should, therefore, be useful in IPAC for SARS-CoV-2, including newly emerging mutational variants^63,64^, future emerging coronaviruses, and other emerging enveloped viruses^23^ (such as Ebola virus, Lassa virus, Nipah virus, and new strains of influenza virus such as the recently emerging G4 genotype H1N1 swine influenza virus^67^ and the variant influenza viruses (H1N1v, H3N2v, H1N2v) in humans)^68^.

## Methods

### Challenge viruses, host cell lines, and reagents

Virucidal efficacy testing against alpha- and beta-coronaviruses was performed for a variety of formulated microbicidal active-containing products per standardized methods. Details on the challenge viruses and their sources and the detector (host) cell lines used for propagation of viral stocks and for cell-based infectivity (titration) assay are displayed in Supplementary Table S1. This table also indicates the culture media used in these assays and the organizations that performed the virucidal efficacy testing.

### Standardized efficacy testing methodologies

Virucidal efficacy evaluations of formulated microbicidal actives against coronaviruses experimentally deposited on a non-porous surface (glass) were conducted per ASTM E1053-20^11^. The active ingredient concentrations, contact times, and exposure temperatures evaluated and the organic soil load are indicated in Table 3. Virucidal efficacy evaluations of formulated microbicidal actives against coronaviruses suspended in liquid matrices were conducted per ASTM E1052-20^12^ or EN 14,476:2013 + A2:2019^13^, depending upon the geographical region in which the formulated microbicide was intended to be marketed. The challenge matrix in each case was cell culture medium containing various organic loads. The active ingredient concentrations in the formulations and the concentrations actually tested (if different), contact times, and exposure temperature evaluated, and the organic soil load, if applicable, are indicated in Tables 4 and 5.

A summary of the standardized methods is presented in Supplemental Materials.

### Calculation of log_10_ reduction, survival half-lives, and time required to reach surface burdens below the MID

Virucidal efficacy data obtained from suspension inactivation and non-porous surface (glass) inactivation studies are presented in terms of log_10_ reduction in titer of the virus, with titers being calculated on the basis of viral cytopathic effect (CPE) (CPE for SARS-CoV-2 in Vero E6 cells is shown in Supplementary Figure S1) and expressed in units of log_10_ tissue culture infectious dose_50_ per mL (TCID_50_/mL).

Survival half-life (t½) of SARS-CoV-2 on experimentally contaminated prototypic HITES, skin, urine, and feces were calculated from data reported in the literature^14–22^. These data consisted of infectious viral titers (log_10_ TCID_50_/mL) measured at various time intervals following drying of the virus on prototypic HITES or skin. Biphasic linear regression plots (log_10_ titer *vs.* time) of the survival data were used to calculate the survival half-lives (t½), as t½ = 0.301/-*m*, where *m* = the slope of the log_10_ titer *vs.* time plots. The time required to reach viral burdens below the human minimal infectious dose (MID) was calculated assuming an initial viral burden of 1 × 10^6^ plaque-forming units (PFU). The time required to reduce the initial viral burden by 1 log_10_ (*D*) was calculated by multiplying the t½ × 3.33 (one t½ = 0.301 log_10_ reduction in titer).

Assuming a human MID for SARS-CoV-2 of ~ 250 PFU (estimated on the basis of mouse infectious dose_50_ values obtained for MHV-1^86^ and SARS-CoV^87^), the time required to bring the burden to 100 PFU was calculated as 4 log_10_ reduction × the time (*D*) required to achieve 1 log_10_ reduction in titer. This calculation was performed as an illustrative example only. It is acknowledged that the selection of an initial viral burden of 1 × 10^6^ PFU is somewhat arbitrary. The latter was based, in part, on estimates of viral particle counts expected to be generated by SARS-CoV-2 by infected persons during loud speaking (> 1 ×  10^3^ virion-containing droplet nuclei per minute)^87^, and the assumption that once generated, the droplet nuclei will eventually settle and contaminate environmental surfaces. The use of ~ 250 PFU as the human MID is a very conservative approach based not on empirical human data, but only on animal (transgenic mouse) studies^87–89^.

## Supplementary Information

Supplementary Information

## Data Availability

All data generated or analyzed during this study are included in this published article (and its Supplementary Information files).
