# Supplementary material for: Microbicidal actives with virucidal efficacy against SARS-CoV-2 and other beta- and alpha-coronaviruses and implications for future emerging coronaviruses and other enveloped viruses
Source: Sci Rep. 2021 Mar 11;11:5626. doi: 10.1038/s41598-021-84842-1 (PMC7952405; doi:10.1038/s41598-021-84842-1)
Supplement: Supplementary file 1 — Supplementary Information [file 41598_2021_84842_MOESM1_ESM.docx]

**Supplemental Material for**

**Microbicidal actives with virucidal efficacy against SARS-CoV-2 and other beta- and alpha-coronaviruses and implications for future emerging coronaviruses and other enveloped viruses**

**M. Khalid Ijaz^1,2 *^, Raymond W. Nims^3^, Sifang Steve Zhou^4^, Kelly Whitehead^1^, Vanita Srinivasan^1^, Tanya Kapes^4^, Semhar Fanuel^4^, Jonathan H. Epstein^5^, Peter Daszak^5^, Joseph R. Rubino^1^, & Julie McKinney^1^**

^1^Reckitt Benckiser LLC, Global Research and Development for Lysol and Dettol, One Philips Parkway, Montvale, New Jersey 07645, USA. ^2^Medgar Evers College of the City University of New York (CUNY), 1650 Bedford Ave, Brooklyn, New York 11225, USA. ^3^RMC Pharmaceutical Solutions, Inc., 1581 Lefthand Circle, Suite A, Longmont, Colorado 80501, USA. ^4^Microbac Laboratories, Inc, 105 Carpenter Drive, Sterling, Virginia 20164, USA. ^5^EcoHealth Alliance, 520 Eighth Avenue, Suite 1200, New York, New York 10018-6507, USA.

Additional detail on standardized methods.

A brief summary of the three standardized methods employed is provided below, with additional details provided in Supplemental Table S1.

ASTM-1052-20 (suspension inactivation): An 0.3-mL aliquot of the challenge viral fluid (virus + soil load) was mixed with 2.7 mL of the test substance by vortex mixing. The reaction mixtures were held for the stipulated contact times under ambient temperature (20±1°C). Following completion of the contact times, the reaction mixtures were quenched by adding a neutralizer to stop the virucidal reaction. The neutralized test samples were passed through a gel filtration column, if required, to reduce cytotoxicity. Neutralized test samples were then serially ten-fold diluted in a dilution medium and inoculated onto host cells to assay for infectious virus using a 50% tissue culture infectious dose (TCID_50_) assay. Briefly, selected dilutions of the neutralized sample were added to cultured host cells (at least six wells per dilution, per reaction mixture) and incubated at 36±2°C with 5±3% CO_2_ for a period of 4 – 9 days. The host cell cultures were observed and refed as necessary, during the incubation period, and were examined microscopically for viral-induced cytopathic effect (CPE).

EN 14476:2013 + A2:2019 (suspension inactivation): A one-mL aliquot of the 10× soil load was mixed with one mL of the challenge virus solution. Eight mL of the test substance, at 125% of the target use concentration or “neat”, were added and the content thoroughly mixed by vortex mixing. A stopwatch was immediately started. The reaction mixtures were held for the stipulated contact times at 20±1°C. Upon completion of the contact times, the reaction mixtures were immediately neutralized by adding an ice-cold neutralizer to stop the virucidal reaction. The neutralized samples were further passed through a gel filtration column, if required, to reduce cytotoxicity to the host cells. Neutralized test samples were then serially ten-fold diluted in a dilution medium and inoculated onto host cells to assay for infectious virus using a 50% tissue culture infectious dose (TCID_50_) assay.

ASTM-E1053-20 (hard surface inactivation): For each test run, an aliquot of 0.4 mL of the challenge viral fluid (virus plus soil load) was added onto a pre-sterilized 10-cm glass Petri dish and spread over the entire surface of the dish. The virus was allowed to dry at ambient temperature. Then 2.0 mL of the test microbicide were added onto the dried viral film by direct pipetting or spray such that the dried virus film was completely covered by the test microbicide. The dishes were held for the stipulated contact times at 20±1°C, then 2.0 mL of neutralizer were added onto the dishes and the viral inoculum/test microbicide/neutralizer mixture was scraped off the dish using a cell scraper. The neutralized test mixture was passed through a gel filtration column, if required, to reduce cytotoxicity to the host cells. The quenched sample was then serially ten-fold diluted in a dilution medium and inoculated onto host cells to assay for infectious virus using the TCID_50_ assay.

| Species^a^ | Genus | Strain | Source | Host Cell | Source | Description | Culture medium |
| --- | --- | --- | --- | --- | --- | --- | --- |
| Human coronavirus | *Alphacoronavirus* | HCoV-229E^b^ | ATCC VR-740 | WI-38 | ATCC CCL-75 | Human lung | MEM + 2% FBS |
| Murine hepatitis virus | *Betacoronavirus* | 1 | ATCC VR-765 | NCTC clone 1469 | ATCC CCL 9.1 | Mouse liver | DMEM + 10% HS |
| SARS-CoV | *Betacoronavirus* | CDC 200300592 | Zeptometrix Corporation | Vero E6 | ATCC CRL-1586 | African green monkey kidney | MEM + 5% FBS |
| MERS-CoV | *Betacoronavirus* | KSA/EMC/2012 | Viroclinics Biosciences B.V. | Vero | Not reported | African green monkey kidney | DMEM + 10% FBS |
| SARS-CoV-2 | *Betacoronavirus* | Isolate USA-WA1/2020 | CDC, through BEI Resources | Vero E6 | ATCC CRL-1586 | African green monkey kidney | MEM + 5% FBS |

**Supplementary Table S1.** Challenge viruses, detector (host) cell lines, and reagents used. ^a^Testing laboratories performing efficacy testing for HCoV-229E: Accuratus Lab Services (Eagan, MN), Microbac Laboratories, Inc. (Sterling, VA); for murine hepatitis virus: Reckitt Benckiser (Montvale, NJ); for SARS-CoV: Microbac Laboratories, Inc. (Sterling, VA); for MERS-CoV: Viroclinics Biosciences B.V. (Rotterdam, The Netherlands); for SARS-CoV-2: Microbac Laboratories, Inc. (Sterling, VA). ^b^Abbreviations used: ATCC, American Type Culture Collection; CDC, U.S. Centers for Disease Control and Prevention; CoV, coronavirus; DMEM, Dulbecco’s minimal essential medium; EMEM, Eagle’s minimal essential medium; FBS, fetal bovine serum; HS, horse serum, MEM, minimal essential medium; MERS, Middle East respiratory syndrome; SARS, severe acute respiratory syndrome.


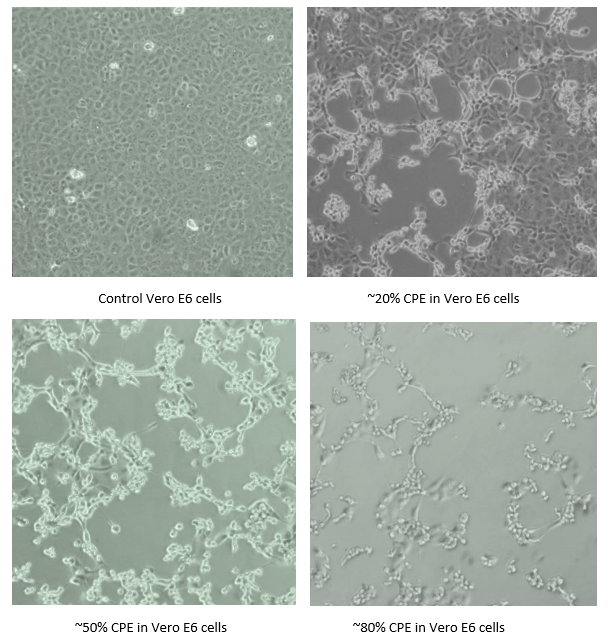


**Supplementary Figure S1.** Appearance and progression of viral cytopathic effect (CPE) in Vero E6 cells inoculated with SARS-CoV-2 and observed over 9 days. Presence or absence of CPE was used to calculate viral titer (log_10_ TCID_50_/mL) during virucidal efficacy testing.
